# Lanthanide-Doped Cs_2_ZrCl_6_ Perovskite Nanocrystals for Multimode Anti-Counterfeiting Application

**DOI:** 10.3390/nano16010068

**Published:** 2026-01-02

**Authors:** Longbin You, Qixin Wang, Yuting Liao, Xiaotian Zhu, Keyuan Ding, Xian Chen

**Affiliations:** College of Materials Science and Engineering, Shenzhen University, Shenzhen 518071, China; 2300341020@email.szu.edu.cn (L.Y.); kyting@szu.edu.cn (K.D.)

**Keywords:** Cs_2_ZrCl_6_ nanocrystals, lanthanide-doped perovskite, multimode emission, anti-counterfeiting

## Abstract

The escalating prevalence of counterfeiting and forgery has imposed unprecedented demands on advanced anti-counterfeiting technologies. Traditional luminescent materials, relying on single-mode or static emission, are inherently vulnerable to replication using commercially available phosphors or simple spectral blending. Multimode luminescent materials exhibiting excitation wavelength-dependent emission offer significantly higher encoding capacity and forgery resistance. Herein, we report the colloidal synthesis of lanthanide-doped Cs_2_ZrCl_6_ nanocrystals (Ln^3+^ = Tb, Eu, Pr, Sm, Dy, Ho) via a robust hot-injection route. These nanocrystals universally exhibit efficient host-to-guest energy transfer from self-trapped excitons (STEs) under 254 nm, yielding sharp characteristic Ln^3+^ f–f emission alongside the intrinsic broadband STE luminescence. Critically, Tb^3+^ enables direct 4f → 5d excitation at ~275 nm, while Eu^3+^ introduces a low-energy Eu^3+^ ← Cl^−^ LMCT band at ~305 nm, completely bypassing STE emission. Due to their multimode luminescent characteristics, we fabricate a triple-mode anti-counterfeiting label displaying different colors under different types of excitation. These findings establish a breakthrough excitation-encoded multimode platform, offering potential applications for next-generation photonic security labels, scintillation detectors, and solid-state lighting applications.

## 1. Introduction

With the rapid expansion of the global commodity economy and the ever-increasing complexity of supply chains, counterfeiting of goods, documents, currencies, and high-value products has evolved into a critical worldwide challenge. Recent years have seen significant focus on developing strategies to prevent counterfeiting [1,2,3,4]. Photoluminescent materials that produce dynamic, multi-dimensional optical signatures under simple UV illumination have emerged as one of the most promising next-generation solutions, offering high encoding capacity, instant visual verifiability, and exceptional forgery resistance [5,6,7,8,9]. Among luminescent materials, lead halide perovskites (LHPs) have attracted immense interest due to their cost-effective solution processability and outstanding optical properties, such as high photoluminescence efficiency, color purity, stability, and tunable emission wavelength [10,11,12,13]. Moreover, LHPs can integrate with flexible material to prepare large-area security patterns through flexible printing techniques, such as screen printing, inkjet printing, and three-dimensional (3D) printing, which has led to the development of anti-counterfeiting and information encryption systems activated by UV light [14,15,16]. However, the toxicity of Pb^2+^ and poor stability of LPHs severely restrict their practical and commercial application [17,18,19].

In this regard, lead-free double perovskites, particularly vacancy-ordered A_2_BX_6_ compounds, have been intensively investigated because they are environmentally friendly and have high stability [20,21,22]. Notably, Cs_2_ZrCl_6_ stands out for its exceptional chemical robustness, negligible toxicity, and intrinsic broadband blue emission (~450 nm) arising from strongly bound self-trapped excitons (STEs) driven by Jahn–Teller distortion of [ZrCl_6_]^2−^ octahedra [23,24]. However, the monochromatic emission of Cs_2_ZrCl_6_ limits its security level, as single-color responses are readily replicated using commercially available phosphors. Doping with metal ions is a well-established approach to tailoring the optical properties of halide perovskites as well as enhancing their stability [25,26]. Among various dopants, trivalent lanthanide ions (Ln^3+^) are particularly attractive owing to due to their narrow, atomic-like 4f–4f emission lines, high color purity, and rich energy levels, which collectively enable tunable luminescence spanning the ultraviolet to near-infrared spectral region [27,28,29]. Fang et al. prepared a serial of Ln^3+^-doped Cs_2_ZrCl_6_ microcrystals, enabling characteristic Tb^3+^ emission, while emissions from other types of Ln^3+^ were not observed [30]. Liu et al. synthesized various Ln^3+^-doped Cs_2_ZrCl_6_ nanocrystals and realized tunable luminescence between blue, green, and red [31]. Li et al. prepared Sb^3+^/Nd^3+^ co-doped Cs_2_ZrCl_6_, which enabled varied color delivery and showed great potential as anti-counterfeiting material [32]. Although multicolor emission under a single excitation wavelength has been achieved, realizing multi-modal luminescence under different excitation wavelengths in Cs_2_ZrCl_6_ remains a challenge.

Herein, we developed a strategy to prepared Ln^3+^-doped (Ln = Tb, Eu, Pr, Sm, Dy, Ho) Cs_2_ZrCl_6_ nanocrystals (NCs) with uniform size and morphology. Ln^3+^ doping into Cs_2_ZrCl_6_ nanocrystals enables efficient STE-to-Ln^3+^ energy transfer, producing lanthanide emission alongside the intrinsic blue STE band. Notably, Tb^3+^ exhibits an intense 4f→5d transition at ~275 nm whereas Eu^3+^ introduces a low-energy Eu^3+^ ← Cl^−^ ligand-to-metal charge-transfer (LMCT) state at ~305 nm. By screen-printing Cs_2_ZrCl_6_:10 mol%Tb^3+^ and Cs_2_ZrCl_6_:10%Eu^3+^ into spatial regions, we fabricated a triple-mode excitation-encoded security label that displayed three different patterns under 254 nm, 275 nm, and 305 nm UV illumination.

## 2. Materials and Methods

### 2.1. Chemicals and Reagents

Terbium acetate hydrate (Tb(OAc)_3_·*x*H_2_O, 99.99%), Europium acetate hydrate (Eu(OAc)_3_·*x*H_2_O, 99.99%) Cerium acetate hydrate (Ce(OAc)_3_·*x*H_2_O, 99.99%), Dysprosium acetate hydrate (Dy(OAc)_3_·*x*H_2_O, 99.99%), Samarium acetate hydrate(Sm(OAc)_3_·*x*H_2_O, 99.99%), Holmium acetate hydrate (Ho(OAc)_3_·*x*H_2_O, 99.99%), chlorotrimethylsilane (TMSCl, 99%), trimethylbromosilane (TMSBr, 99%), 1-octadecene (1-ODE,90%), oleylamine (OLA, 70%), and oleic acid (OA, 90%) were purchased from Sigma-(Shanghai, China). Cesium acetate (Cs(OAc), 99.99%), Praseodymium acetate hydrate (Pr(OAc)_3_·*x*H_2_O, 99.9%) was purchased from J&K Scientific Ltd., Beijing, China. Zirconium carbonate oxide (CO_4_Zr,99%) was purchased from Macklin Biochemical Co. Ltd., *Shanghai, China. Chloroform (CHCl_3_, 99%) was purchased from Guangshi Reagent Co. Ltd., Guangzhou, China. All chemicals were used as received without further purification.

### 2.2. Synthesis of Cs_2_ZrCl_6_ and Ln^3+^-Doped Cs_2_ZrCl_6_ Nanocrystals

Cs_2_ZrCl_6_ NCs were synthesized through a modified hot-injection method. In a typical procedure, 272 mg (1.42 mmol) of CsOAc and 123.7 mg (0.71 mmol) of CO_4_Zr were added to a 50 mL three-neck flask containing 10 mL of 1-ODE, 2.8 mL of oleic acid and 615 μL of oleylamine. The mixture was degassed and dried under a vacuum for at 110 °C for 30 min to obtain a clear, transparent solution. The mixture was then heated to 220 °C under an Ar atmosphere. Upon reaching this temperature, 400 μL TMSCl was rapidly injected. After reaction for about 45 s, the obtained crude solution was cooled in an ice-water bath. The resulting NCs were isolated by centrifugation at 7850 rpm for 5 min and the supernatant was discarded. The precipitate was redispersed in 4 mL of chloroform through vigorous vortexing to form a stable colloidal dispersion, followed by the addition of 8 mL ethanol to induce precipitation. The mixture was then centrifuged, and the supernatant was discarded. This washing procedure was repeated twice. Finally, the cleaned product was redispersed in 4 mL of chloroform for storage.

Ln^3+^-doped Cs_2_ZrCl_6_ NCs (Ln = Tb, Eu, Dy, Pr, Sm, Ho) were prepared following the same procedure by adding the corresponding lanthanide (III) acetate hydrate.

### 2.3. Materials’ Characterizations

X-ray diffraction (XRD) analysis was carried out on a MiniFlex600 X-ray diffractometer with Cu Kα radiation λ = 1.5406 Å (Rigaku Corporation, Tokyo, Japan). Transmission electron microscope (TEM) images were acquired using a HT7700 tungsten filament transmission electron microscope (Hitachi High-Tech Corporation, Tokyo, Japan). High-resolution TEM images and energy-dispersive X-ray spectroscopy (EDS) mapping were recorded on an F200 field emission transmission electron microscope equipped with an EDS attachment (JEOL). Photoluminescence spectra were acquired using an FS5 Transient spectrometer (Edinburgh Instruments Ltd., Livingston, UK). Fluorescence decay curves and photoluminescence quantum yields were recorded on an FLS1000 fluorescence spectrophotometer (Edinburgh Instruments Ltd., Livingston, UK).

### 2.4. Fabrication of Security Pattern

The security patterns were prepared by using Cs_2_ZrCl_6_: 10 mol%Tb^3+^ and red Cs_2_ZrCl_6_: 10 mol%Eu^3+^ NCs. Luminescent inks were prepared by mixing the PDMS glue and curing agent at a mass ratio of 10:1. Subsequently, the Cs_2_ZrCl_6_: 10 mol%Tb^3+^ (or Cs_2_ZrCl_6_: 10 mol%Eu^3+^) phosphor were added to the PDMS mixture at a mass ratio of 3:2 and vigorously stirred for 5 h to ensure homogeneous dispersion. The resulting mixture was then screen-printed onto polypropylene (PP) substrates using custom-designed stencils. After printing, the patterns were cured at 80 °C for 10 min. Photographs of the security patterns under 254 nm, 275 nm, and 305 nm excitation were recorded in a dark chamber with handheld UV lamps.

## 3. Results and Discussion

### 3.1. Structural and Morphological Properties of Tb^3+^-Doped Cs_2_ZrCl_6_ NCs

**Figure 1:**
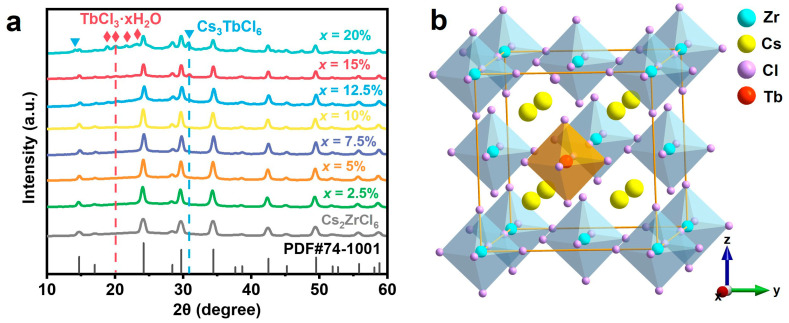
(**a**) Powder XRD patterns of Cs_2_ZrCl_6_:*x*Tb^3+^ NCs. The diffraction peaks marked with diamonds and triangles correspond to TbCl_3_·*x*H_2_O and Cs_3_TbCl_6_, respectively. (**b**) Schematic of the crystal structure of Cs_2_ZrCl_6_:Ln^3+^ NCs.

Powder X-ray diffraction (XRD) patterns reveals that samples doped with Tb^3+^ below 10 mol% retain the pure cubic vacancy-order double-perovskite structure of Cs_2_ZrCl_6_ (space group Fm$\bar{3}$m, PDF#74-1001), with no detectable impurity phase (Figure 1a). The host lattice consists of isolated [ZrCl_6_]^2−^ octahedra alternatively arranged with ordered vacancies and separated by Cs^+^ cation (Figure 1b) [33]. Given the close ionic radii and identical octahedral coordination preference of Tb^3+^ (r ≈ 0.92 Å, CN = 6) and Zr^4+^ (r = 0.72 Å, CN = 6), Tb^3+^ ions preferentially substitute at Zr^4+^ sites, forming [TbCl_6_]^3−^ units, instead of substitution at the larger Cs^+^ site (r = 1.68 Å, CN = 8) [31,34,35]. The resulting charge imbalance from this aliovalent substitution is typically compensated by the formation of chloride vacancies to preserve lattice electroneutrality [26]. When the concentration of Tb^3+^ reaches 10 mol%, weak additional diffraction peaks emerge in the 20° range, which is attributable to trace TbCl_3_·*x*H_2_O. Upon further increasing the Tb^3+^ concentration to 12.5 mol%, more extraneous peaks emerged, showcasing the formation of Cs_3_TbCl_6_. This phase segregation is attributed to the combined effects of ionic radius mismatch between Tb^3+^ and Zr^4+^ ions, which collectively induce lattice strain and thermodynamic instability beyond ~10 mol% doping. This solubility limit arises from cumulative lattice strain induced by ionic radius mismatch and electrostatic imbalance between Tb^3+^ and Zr^4+^; collectively, these factors destabilize the solid-solution formation and drive phase segregation at higher doping levels [31,34].

**Figure 2:**
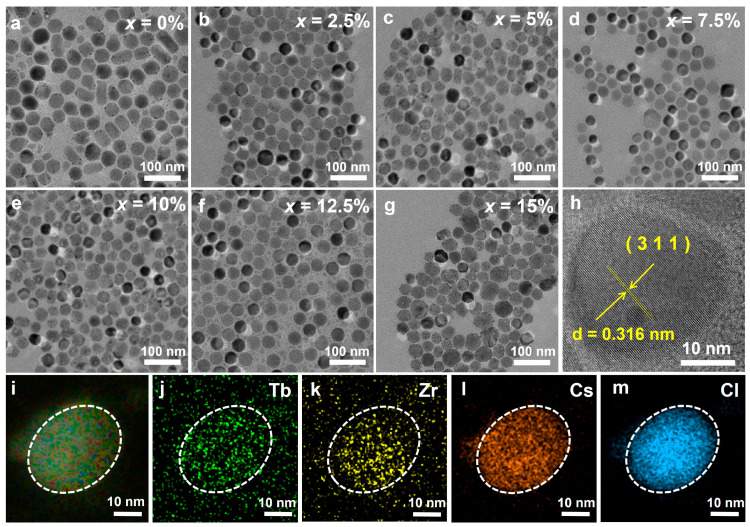
(**a**–**g**) TEM images of Cs_2_ZrCl_6_: *x*Tb^3+^ (*x* = 0–15 mol%) NCs. (**h**) High-resolution TEM image of a single Cs_2_ZrCl_6_:Tb^3+^ NCs. (**i**–**m**) Elemental mappings of Tb, Zr, Cs and Cl for Cs_2_ZrCl_6_:Tb^3+^ NC.

Transmission electron microscopy (TEM) images show that Cs_2_ZrCl_6_:*x*Tb^3+^ NCs exhibited quasi-spherical, prism-like, and polygonal morphologies, with most having sizes of around 30 nm (Figure 2a–g). Upon Tb^3+^ doping, the number of prism-like particles decreases, likely because Tb^3+^ alters surface energy and growth kinetics [26]. All the prepared NCs exhibit excellent dispersion, regular morphology, and uniform size. The high-resolution transmission electron microscope (HRTEM) image shows clear lattice fringes, indicating the single-crystalline nature of the Cs_2_ZrCl_6_ nanocrystals (Figure 2h). The observed d-spacing of lattice fringes is ~0.316 nm, which is in good agreement with the lattice spacing of (311) planes of Cs_2_ZrCl_6_ (PDF#741001). Furthermore, energy-dispersive X-ray spectroscopy (EDS) elemental mapping demonstrates the homogeneous elements distributions of Cs, Zr, Tb and Cl through the individual Cs_2_ZrCl_6_: 10 mol% Tb^3+^ NCs (Figure 2i–m), further confirming the successful doping of Tb^3+^ ions into the Cs_2_ZrCl_6_ host matrix.

**Figure 3:**
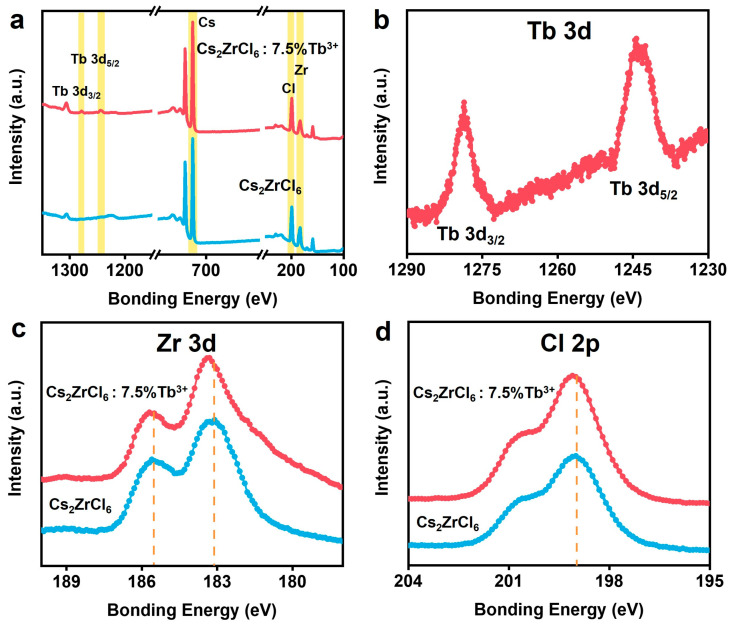
(**a**) Wide XPS spectrum of Cs_2_ZrCl_6_:Tb^3+^. (**b**–**d**) High-resolution XPS spectra of Tb, Zr and Cl.

X-ray photoelectron spectroscopy (XPS) measurement was performed to further explore the valence state and chemical composition of ions in Cs_2_ZrCl_6_ nanocrystals with and without Tb^3+^ doping (Figure 3a). With the doping of Tb^3+^, two peaks corresponding to Tb were observed. Furthermore, the two peaks at 1278 and 1243 eV were attributed to Tb 3d_3/2_ and Tb 3d_5/2_, respectively (Figure 3b), as well as two peaks at ~182.7 and ~185.1 eV which were attributed to Zr 3d_5/2_ and Zr 3d_3/2_, respectively (Figure 3c). A slight blue shift was observed in the Zr 3d and Cl 2p XPS peaks of Tb^3+^-doped Cs_2_ZrCl_6_ nanoparticles (Figure 3c,d). This was due to the charge imbalance induced by Tb^3+^ substituting Zr^4+^, which is consistent with prior reports on aliovalent doping in halide perovskites [36,37].

### 3.2. Optical Properties of Ln^3+^-Doped Cs_2_ZrCl_6_ NCs

**Figure 4:**
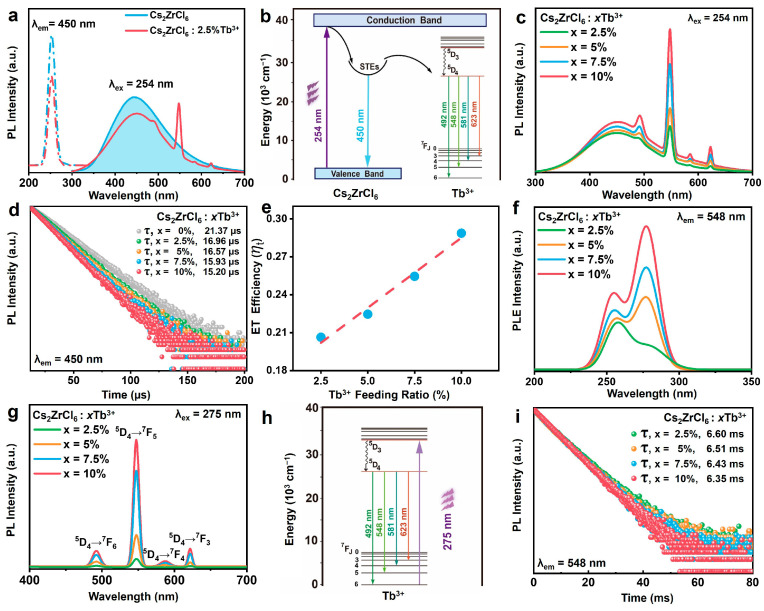
(**a**) PLE (dashed lines) and PL (solid lines) spectra of undoped (blue) and 10% Tb^3+^ doped Cs_2_ZrCl_6_ NCs (red). (**b**) The photoluminescence mechanism diagram of Cs_2_ZrCl_6_: Tb^3+^ NCs excited under 254 nm. (**c**) PL spectra of Cs_2_ZrCl_6_: (2.5–10%) Tb^3+^ NCs excited by 254 nm. (**d**) The decay curves of Cs_2_ZrCl_6_: (2.5–10 mol%) Tb^3+^ NCs monitored at 450 nm. (**e**) The ET efficiency (*η*t) variation of Cs_2_ZrCl_6_: (2.5–10 mol%) Tb^3+^ NCs (monitored at 450 nm) as a function of the Tb^3+^ feeding ratio. The dashed line is the linear regression of the measured data. (**f**) PLE spectra of Cs_2_ZrCl_6_: (2.5–10 mol%) Tb^3+^ NCs monitored at 548 nm. (**g**) PL spectra of Cs_2_ZrCl_6_: (2.5–10 mol%) Tb^3+^ NCs in the visible region excited by 275 nm. (**h**) The photoluminescence mechanism diagram of Cs_2_ZrCl_6_: xTb^3+^ NCs excited at 275 nm. (**i**) The decay curves of Cs_2_ZrCl_6_: (2.5–10 mol%) Tb^3+^ NCs monitored at 548 nm.

The optical properties of as-prepared Cs_2_ZrCl_6_ NCs doped with Tb^3+^ ions were systematically investigated. The PLE spectrum of pristine Cs_2_ZrCl_6_ NCs monitored at 450 nm exhibits a sharp excitation peak located at 254 nm, which is attributed to the Zr–Cl ligand-to-metal charge transfer transition (LMCT) in [ZrCl_6_]^2-^ complex clusters (Figure 4a) [38,39]. Such charge transfer transitions were widely observed not only in metal oxide complexes like [VO_4_]^3−^ but in other perovskite-structured materials such as Cs_2_HfCl_6_ [40,41]. Upon the excitation at 254 nm, undoped Cs_2_ZrCl_6_ NCs exhibited a broad emission peak centered at 450 nm (Figure 4a). This broad emission of the matrix was ascribed to intrinsic STEs of the host in consequence of the strong electron–phonon coupling in metal halides with a soft lattice [42,43,44,45]. For Tb^3+^-doped Cs_2_ZrCl_6_ NCs, the host STE emission (~450 nm) sharply declined, accompanied by the emergence of intense 492 nm, 548 nm, 581 nm and 623 nm under the excitation at 254 nm (Figure 4a). These discrete peaks are assigned to the ^5^D_4_ → ^7^F_J_ (J = 6, 5, 4, 3) intra-configurational transitions of Tb^3+^ ions driven by efficient energy transfer from STEs to Tb^3+^ ions (Figure 4b). Under 254 nm excitation, electrons were promoted from the valence band to the conduction band, followed by non-radiative relaxation into self-trapped states within distorted [ZrCl_6_]^2−^ octahedra, yielding the characteristic broad blue STE emission. In the presence of Tb^3+^, a fraction of this trapped energy resonantly transferred to the Tb^3+^ 5d manifold via Förster–Dexter exchange [32,44], followed by rapid internal conversion to the ^5^D_4_ level and subsequent radiative decay to the ^7^Fⱼ ground states, producing sharp Tb^3+^ emission lines. Thus, under 254 nm excitation, Tb^3+^ luminescence was primarily sensitized by host STEs rather than direct dopant excitation, highlighting the critical role of the host-to-guest energy channel in enabling tunable emission color.

To shed more light on the optical properties of Cs_2_ZrCl_6_: xTb^3+^ NCs, Cs_2_ZrCl_6_ with Tb^3+^ doping concentrations ranging from 2.5 to 10 mol% was investigated. As the Tb^3+^ concentration increased, the Tb^3+^ characteristic emission gradually enhanced. Remarkably, the STE emission band at ~450 nm recovered slightly in intensity rather than exhibiting the anticipated decrease typically observed in conventional donor–acceptor systems (Figure 4c). The enhancement of host STE emission is ascribed to Tb^3+^-induced lattice expansion upon substitution of the smaller Zr^4+^. This structural relaxation exacerbates the Jahn–Teller distortion of the [ZrCl_6_]^2−^/[TbCl_6_]^3−^ octahedra in the excited state, thereby deepening the self-trapping potential and ultimately enhancing the radiative efficiency of STE recombination [42,46,47].

To further understand the ET process from host STEs to Tb^3+^ ions, PL decay curves of Cs_2_ZrCl_6_: xTb^3+^ NCs (x  = 0~10 mol%) were recorded at 450 nm under 254 nm excitation (Figure 4d). The effective lifetimes were determined by [48]

$$\tau_{eff}=\frac{1}{I_{0}}\int_{0}^{\infty }I(t)dt$$

where *I*_0_ and *I*(*t*) represent the maximum luminescence intensity and luminescence intensity at time *t* after cutting off the excitation light, respectively. Upon Tb^3+^ doping, the effective STE lifetime decreased from ~21.37μs to ~15.20 μs, further demonstrating the non-radiative energy transfer from the host to Tb^3+^. The progressive acceleration of decay with increasing Tb^3+^ concentration confirms that ET is enhanced at higher dopant concentrations, consistent with reduced average donor–acceptor separation. The ET efficiency (*η*t) was adopted and can be calculated by the following equation [32]:

$$\eta_{t}=1-\frac{\tau_{X}}{\tau_{s}}$$

where $\tau_{X}\;$ and $\tau_{s}$ are the lifetimes of STEs in the presence or absence of Tb^3+^ ions, respectively. Figure 4e shows that the ET efficiency ($\eta_{t}$) from host STEs to Tb^3+^ increases gradually with the Tb^3+^ doping concentration, reaching a maximum (ca. 28%) when Tb^3+^ feeding concentrations are at 10%. The ET efficiency of Ln^3+^-doped Cs_2_ZrCl_6_ NCs is obviously higher than that of Cs_2_ZrCl_6_ microcrystals (ca. 20%). Furthermore, the ET course in perovskite doped with weak-emission Ln^3+^ ions cannot be detected in Cs_2_ZrCl_6_ MCs, but Cs_2_ZrCl_6_ NCs can, indicating that nanocrystals are more advantageous when it comes to studying the ET process in perovskite doped with Ln^3+^ ions [30].

The excitation spectra monitored at 548 nm (^5^D_4_ → ^7^F_5_ transition) reveals, in addition to the host STE band at 254 nm, a distinct new excitation peak centered at 275 nm in Tb^3+^-doped Cs_2_ZrCl_6_ NCs. This 275 nm band is absent in undoped samples and scales linearly with the Tb^3+^ concentration (Figure 4f), confirming its origin from f–d transition of Tb^3+^ ions (Figure 4h). Excitation wavelength-dependent PL spectra further demonstrate two independent sensitization channels. When Cs_2_ZrCl_6_: xTb^3+^ NCs were excited by 275 nm, only the feature emission of Tb^3+^ ions existed without the host STEs emission. As the Tb^3+^ doping concentration increased, the intensity of characteristic emission of Tb^3+^ ions was augment little by little, as illustrated in Figure 4g. The Tb^3+^ lifetimes monitored at 548 nm show only slight shortening as the Tb^3+^ doping concentration increases (Figure 4i). This behavior arises from the vacancy-ordered structure of Cs_2_ZrCl_6_, which features isolated [ZrCl_6_]^2−^/[TbCl_6_]^3−^ octahedra separated by Cs^+^ cations and ordered vacancies. Such spatial confinement effectively isolates individual Tb^3+^ dopants, minimizing energy migration between sites and suppressing concentration quenching through cross-relaxation or non-radiative trapping [49,50].

**Figure 5:**
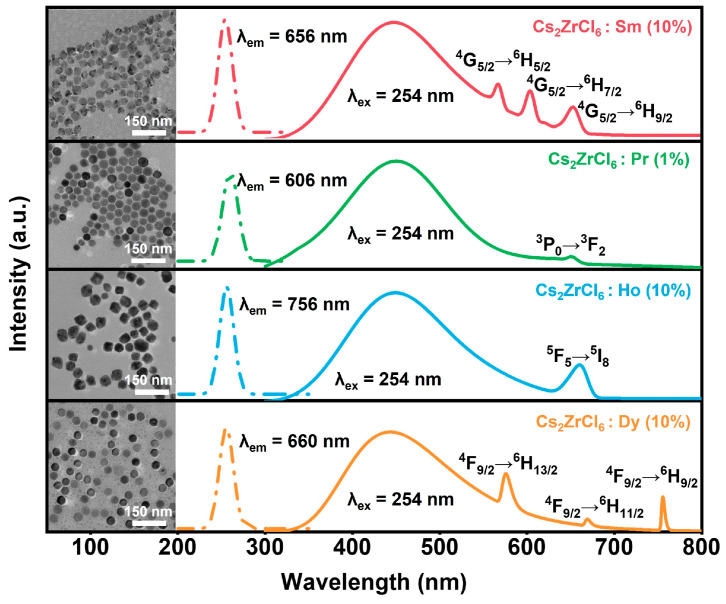
PLE (dashed lines) and PL (solid lines) spectra of Cs_2_ZrCl_6_:Ln^3+^ (Ln = Pr, Sm, Dy, Ho) NCs. Inset: TEM images of Cs_2_ZrCl_6_:Ln^3+^ (Ln = Pr, Sm, Dy or Ho) NCs.

To assess the universality of host-sensitized lanthanide luminescence in Cs_2_ZrCl_6_ NCs, a series of additional Ln^3+^ (Ln = Pr, Sm, Dy, Ho) ion-doped Cs_2_ZrCl_6_ NCs were prepared using the identical hot injection protocol. Based on the XRD results for Tb doping, impurity phases of Cs_3_LnCl_6_ form when the Ln^3+^ concentration exceeds 10 mol%. Therefore, the doping level for Sm^3+^, Dy^3+^, and Ho^3+^ is set at 10 mol%. For Pr^3+^, the concentration is reduced to 1 mol% due to its pronounced susceptibility to concentration quenching. TEM images confirm that all Ln^3+^-doped Cs_2_ZrCl_6_ nanocrystals retain highly uniform morphology and an average particle size of ~30 nm (inset of Figure 5). PLE spectra monitored for the respective Ln^3+^ emissions reveal that all these Ln^3+^-doped samples exhibit a dominant excitation peak at 254 nm, corresponding to the host Zr^4+^ ← Cl^−^ ligand-to-metal charge-transfer (LMCT) transition. Under 254 nm excitation, all the samples exhibiting composite emission spectra comprise a broad blue STE band centered at ~450 nm and well-resolved f-f transition emission of the respective Ln^3+^ activators (Figure 5). The consistent observation of Ln^3+^-specific f–f emission across this diverse set of Ln^3+^ ions provides compelling evidence for efficient and universal STE-to-Ln^3+^ energy transfer in the Cs_2_ZrCl_6_ NC, enabling tunable multicolor luminescence via a single broadband sensitizer.

**Figure 6:**
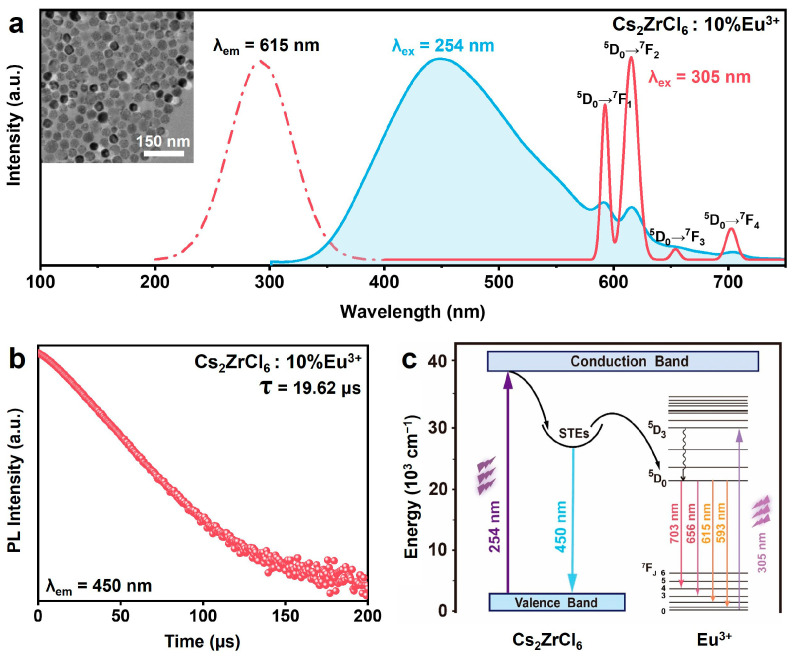
(**a**) PLE (dashed lines) and PL (solid lines) spectra of 10 mol% Eu^3+^-doped Cs_2_ZrCl_6_ NC. Inset: TEM image of Cs_2_ZrCl_6_:10 mol% Eu^3+^ NCs. (**b**) The photoluminescence mechanism diagram of Cs_2_ZrCl_6_: xEu^3+^ NCs excited at 254 nm and 305nm. (**c**) The decay curves of Cs_2_ZrCl_6_: 10 mol% Eu^3+^ NCs monitored at 450 nm.

Compared to Dy^3+^-, Sm^3+^-, Pr^3+^-, or Ho^3+^-doped Cs_2_ZrCl_6_ NCs, the excitation spectrum of Cs_2_ZrCl_6_: 10 mol% Eu^3+^ NCs monitoring at 615 nm (^5^D_0_ → ^7^F_2_ transition of Eu^3+^) shows a pronounced redshift of the dominant excitation peak from 254 nm to a wide band centered at 305 nm (Figure 6a). The redshift is attributed to the formation of a low-energy Eu^3+^ ← Cl^−^ LMCT state that lies below the intrinsic Zr^4+^ ← Cl^−^ LMCT band of the host lattice. The host LMCT at 254 nm remains detectable and the STE emission is accompanied by weak Eu^3+^ f–f lines, indicative of a partial STE-to-Eu^3+^ energy transfer under 254 nm excitation (Figure 6c). A decay curve monitored at 450 nm shows a lifetime of ~19.62 μs for Cs_2_ZrCl_6_: 10 mol% Eu^3+^ (Figure 6b), which is slightly shorter than that of undoped Cs_2_ZrCl_6_, confirming the energy transfer from STEs to Eu^3+^ ions. The transfer efficiency is about ~8.2%, lower than that observed in Tb^3+^-doped Cs_2_ZrCl_6_. Notably, the Eu^3+^ ← Cl^−^ CT state becomes the dominant excitation pathway for Eu^3+^ luminescence. Notably, 305 nm excitation selectively populates the Eu^3+^-centered CT state, resulting in pure sharp Eu^3+^ f–f transition emission lines with complete suppression of the host STE broadband emission (~450 nm).

### 3.3. Anti-Counterfeit Application of Ln^3+^-Doped Cs_2_ZrCl_6_ NCs

**Figure 7:**
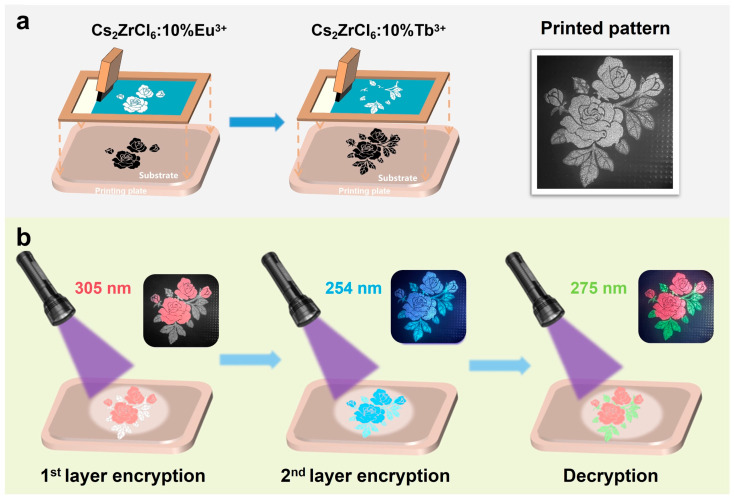
(**a**) Schematic of multicolor pattern preparation through serial screen printing and a photograph of the pattern under ambient light. (**b**) Optical photographs of the prepared pattern under 295 nm, 254 nm, and 275 nm UV lamp excitation, respectively.

To exploit the distinct excitation wavelength-dependent luminescence of Tb^3+^- and Eu^3+^-doped Cs_2_ZrCl_6_ nanocrystals, we fabricated a multimode optical security pattern by spatially integrating Cs_2_ZrCl_6_:10 mol% Tb^3+^ and Cs_2_ZrCl_6_:10 mol% Eu^3+^ phosphors in different regions within a flexible composite matrix (Figure 7a). The pattern was produced via sequential screen-printing onto a polypropylene (PP) substrate, employing luminescent inks prepared by dispersing the nanocrystals in polydimethylsiloxane (PDMS) at a phosphor-to-PDMS mass ratio of 3:2. Specifically, the flower region was first printed using a Cs_2_ZrCl_6_:10%Eu^3+^@PDMS ink, followed by printing of the leaf region with Cs_2_ZrCl_6_:10%Tb^3+^@PDMS ink.

The resulting security label exhibits three fully orthogonal luminescence responses depending solely on the excitation wavelength (Figure 7b). Under 305 nm excitation, corresponding to the low-energy Eu^3+^ ← Cl^−^ LMCT state, only the flower region emits intense red light, dominated by the Eu^3+ 5^D_0_ → ^7^F_1_ and ^7^F_2_ transition at 595 and 611 nm. Meanwhile, the leaf region remains dark due to negligible absorption by either the Cs_2_ZrCl_6_ host bandgap or the Tb^3+^ transitions in this wavelength range. At 254 nm excitation, both the leaf and flower region display blue emission (~450 nm) arising from STE recombination within the Cs_2_ZrCl_6_ host, resulting in complete pattern visualization in a single color. Under 275 nm excitation, the flower region exhibits dominant red emission from Eu^3+^, whereas the leaf region emits green light arising from f-f transition of Tb^3+^ ions. This triple-mode, excitation wavelength-encoded behavior renders replication difficult and prohibitive using conventional phosphor blending, significantly enhancing the security level of the anti-counterfeiting feature.

## 4. Conclusions

In summary, we have established a robust and reproducible hot-injection protocol for the synthesis of Ln^3+^-doped Cs_2_ZrCl_6_ nanocrystals (Ln = Tb, Eu, Pr, Sm, Dy, Ho) that delivers uniform size and morphology. Systematic spectroscopic investigations reveal universal and efficient energy transfer from STEs to Ln^3+^ activators. Tb^3+^-doped nanocrystals exhibit two independent sensitization routes: host LMCT excitation at 254 nm produces composite STE and Tb^3+^ emission, whereas direct 4f → 5d absorption at 275 nm yields pure green Tb^3+^ luminescence. Eu^3+^ doping introduces a low-energy Eu^3+^ ← Cl^−^ LMCT band at ~305 nm, enabling pure f–f transition emission of Eu^3+^ without host STE luminescence. We demonstrate an advanced triple-mode, excitation wavelength-dependent anti-counterfeiting technology based on Cs_2_ZrCl_6_:10 mol% Tb^3+^ and Cs_2_ZrCl_6_:10 mol% Eu^3+^ NCs. This present work establishes Cs_2_ZrCl_6_ nanocrystals as a versatile, broadband-sensitizing host for lanthanide luminescence, offering excitation-tunable multicolor emission for next-generation photonic security labels, scintillation detectors, and solid-state lighting applications.

## Data Availability

All data are available in the manuscript.

## Abbreviations

The following abbreviations are used in this manuscript:

PL	Photoluminescence.
PLE	Photoluminescence.
UV	Ultraviolet.
NCs	Nanocrystals.
MCs	Microcrystals.
LHP	Lead halide perovskites.
STEs	Self-trapping excitons.
TEM	Transmission electron microscopy.
HRTEM	High-resolution transmission electron microscopy.
XRD	X-ray diffraction.
EDS	Energy-dispersive spectrometer.
XPS	X-ray photoelectron spectroscopy.
SEM	Scanning Electron Microscope.
ET	Energy transfer.
PDMS	Polydimethylsiloxane.
